# Evaluation of In Vivo Biological Activity Profiles of Isoindole-1,3-dione Derivatives: Cytotoxicity, Toxicology, and Histopathology Studies

**DOI:** 10.1021/acsomega.3c00560

**Published:** 2023-03-21

**Authors:** Aytekin Köse, Meltem Kaya, Canberk Tomruk, Yiǧit Uyanıkgil, Nurhan Kishalı, Yunus Kara, Gülşah Şanlı-Mohamed

**Affiliations:** † Department of Chemistry, Faculty of Science and Letters, Aksaray University, Aksaray 68100, Turkey; ‡ Department of Chemistry, Faculty of Science, İzmir Institute of Technology, İzmir 35430, Turkey; § Department of Histology and Embryology, Faculty of Medicine, Ege University, İzmir 35100, Turkey; ∥ Department of Chemistry, Faculty of Science, Atatürk University, Erzurum 25240, Turkey

## Abstract

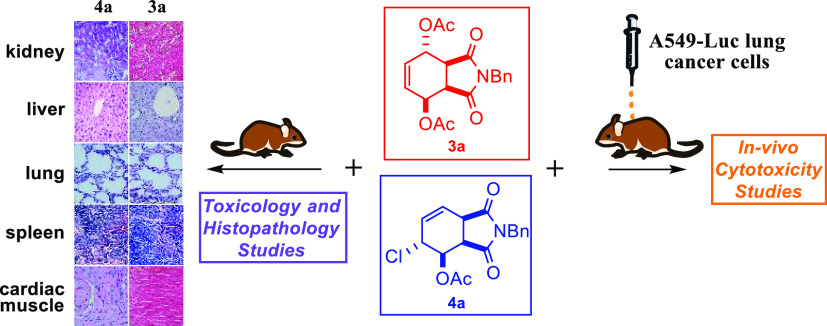

The anticancer activity of *N*-benzylisoindole-1,3-dione derivatives was evaluated against adenocarcinoma (A549-Luc). First, 3-(4,5-dimethylthiazol-2-yl)-2,5-diphenyltetrazolium bromide activity assay studies of two isoindole-1,3-dione derivatives were performed against A549 cell lines. Both compounds showed inhibitory effects on the viability of A549 cells. Then, we explored the potential of these compounds as active ingredients by in vivo studies. Nude mice were given A549-luc lung cancer cells, and tumor growth was induced with a xenograft model. Then, nude mice were divided into three groups: the control group, compound 3 group, and compound 4 group. After application of each compound to the mice, tumor sizes, their survival, and weight were determined for 60 days. Furthermore, toxicological studies were performed to examine the effects of the drugs in mice. In addition to toxicological studies, histopathological analyses of organs taken from mice were performed, and the results were evaluated. The obtained results showed that both N-benzylisoindole derivatives are potential anticancer agents.

## 1 Introduction

**Figure 1:**
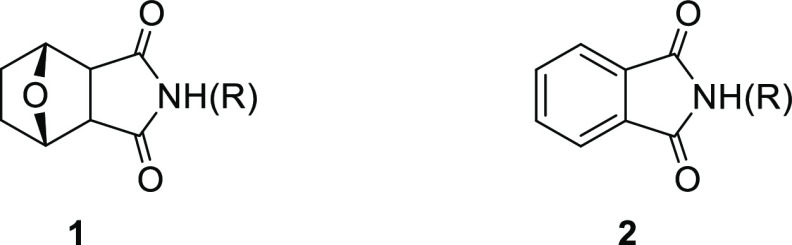
Structure of *N*-derivative norcantharimide and phthalimide or isoindole-1,3-dione.

Norcantharimides (**1**), which are derivatives of isoindole-1,3-dione (**2**), have aroused considerable attention due to their potential anticancer effects. They also have inhibitory effects against protein phosphatase 1 and 2A (PP1 and 2A).^1−3^ Thus, the synthesis of isoindole-1,3-dione derivatives has been of great interest to many organic and pharmaceutical chemists (Figure 1).

**Figure 2:**
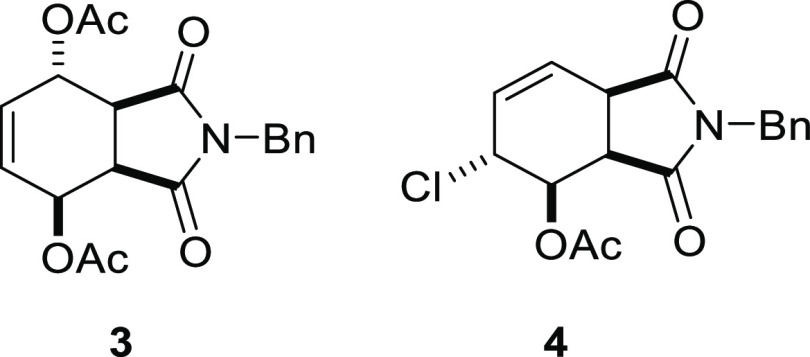
Structures of diacetoxy/chloroacetoxy *N*-benzylisoindole-1,3-dione derivatives.

The anticancer effects of some synthesized isoindole-1,3-dione derivatives against numerous cancer cell lines have been investigated. For example, McCluskey and colleagues synthesized various norcantharimides and examined their cytotoxicity against different cancer cell lines. They reported the effects of differences in skeletal structure on the anticancer activity of the compounds.^4^ Pen-Yuan et al. also investigated the synthesis and anticancer activity of the N-substituted cantharimides (aliphatic, aryl, and pyridyl groups) in vitro against HepG2 and HL-60 cells.^5^ Kok et al. reported the synthesis and cytotoxicity of some cantharimide derivatives.^6^ They have thoroughly explored the electronic properties of the functional group on the cytotoxicity of some cantharimide derivatives. The tumor inhibitory influence of *N*-methylcantharimide has been explored in animals.^7,8^ Recently, we have studied a versatile synthetic approach to the synthesis of new norcantharimide derivatives.^9−12^ We reported the antiproliferative properties of some of the isoindole-1,3-dione derivatives against different cell lines like MCF-7, A549, HeLa, and HT-29 cell lines. More recently, in particular, we reported the synthesis of diacetoxy and chloroacetoxy substituted isoindole derivatives and their inhibitory effects on the viability of HeLa cells.^9^ The best cytotoxic activity was determined in *N*-benzyl isoindole derivatives **3** and **4** (Figure 2).

On the other hand, it is determined in our previous studies that a compound has cytotoxic activity against more than one cell lines.^10−12^ In this context, we investigated whether *N-*benzyl derivatives, which are active against HeLa cell lines, are also active against A549 cell lines, and we determined that the degree of cytotoxicity in A549 cell lines increased compared to HeLa cell lines (Table 1).

**Table 1:** IC_50_ Values of Compounds **3** and **4**

entry	time (h)	compound **4** (**X**) (IC_50_ μM)	compound **3** (**Y**) (IC_50_ μM)
1.A549 cell lines	48	116.26	114.25
2.HeLa cell lines	48	140.60	148.59

Considering these results, we decided to conduct preclinical (animal experiments) studies to evaluate the anti-cancer potential of *N*-benzyl derivatives against adenocarcinoma (A549-Luc) cells. Herein, we report the evaluation of in vivo therapy of *N*-benzyl isoindole derivatives against adenocarcinoma (A549-Luc) cells.

## 2 Materials and Methods

The substances were obtained from Dr. Kara’s lab and purified by crystallization. The structure and individuality of the obtained substances were proved by nuclear magnetic resonance (NMR) analysis and elemental analysis.^9^

### 2.1 Cell Cultures

The A549 (human lung carcinoma) cell line was grown in a Dulbecco’s Modified Eagle Medium (DMEM) containing 10% fetal bovine serum (FBS) and 100 units/mL penicillin–100 μg/mL streptomycin and 2 mM l-glutamine for cytotoxicity experiments. Cells were maintained at 37 °C temperature conditions containing 5% CO_2_. When the cells covered 95–98% of the flask, they were passaged.

Luc cell line (A549-Luc cell), which has luciferase activity, was used to determine the cancer treatment potential of compounds **3** and **4** for in vivo experiments. Cells were cultured in DMEM Ham’s F12 [10% FBS, 1% l-glutamine, 1% gentamicin, and 1 mM 4-(2-hydroxyethyl)-1-piperazineethanesulfonic acid] broth in a CO_2_ incubator set at 37 °C. Sufficient stocks were prepared by passaging the cells twice a week, and cells that actively proliferated in the logarithmic phase were used in the tests.

### 2.2 In Vitro Cytotoxicity Assay

The 3-(4,5-dimethylthiazol-2-yl)-2,5-diphenyltetrazolium bromide (MTT) proliferation assay was performed to evaluate the cytotoxicity of the **3** and **4** compounds. Cells were seeded at a density of 5 × 10^4^ cells/cm^2^ and incubated at 37 °C in 95% air, 5% CO_2_ environments for 24 h. Substances **3** and **4** were first dissolved in DMSO, then dilutions were prepared by dispersing them in the DMEM cell growth medium. The dose intervals of substances **3** and **4** were prepared as 400, 200, 100, 50, and 25 μM. The dose intervals of substances **3** and **4** were placed on a 24-well cell culture plate and then incubated for 24 h. After incubation, the medium was removed and replaced with the MTT-containing medium. Plates were incubated for an additional 4 h at 37 °C. MTT medium was removed, and 100 μL of DMSO was added to dissolve the formazan crystals. The absorbance was determined using a plate reader at a wavelength of 540 nm. IC_50_ values for 24, 48, and 72 h were calculated using GraphPad Prism5 software (GraphPad, San Diego, CA).

### 2.3 In Vivo Monitoring of Cancer Treatment Potential

Approval for monitoring the cancer treatment potential of the synthesized structures in vivo conditions was taken by the Animal Ethics Committee of Ege University on 24.01.2018 with the number 2018-015. Male atypical nude mice, 6–8 weeks old, were used for in vivo xenograft tumor models. In order to determine the lung cancer treatment potential of the synthesized **3** and **4** substances, A549-Luc lung cancer cells were given to nude mice and tumor growth was achieved with the xenograft model. For the purpose of xenograft administration, nude mice were injected subcutaneously with 100 μL of 5 × 10^6^ A549-Luc cell suspension. The presence of cancer after cell administration to nude mice was followed by imaging with an IVIS device. In order to monitor tumor formation and tumor volume detection, luciferin [Biovision Inc., USA; 12 mg/mL in phosphate-buffered saline (PBS)] was injected intraperitoneally followed by visualization with IVIS 5 min later. When tumor volume reaches about 2000 mm^3^, cancerous nudes are divided into three groups: the control group, **3** group, and **4** group. Grouping was made as 3–5 animals in each group. The following procedures were prepared according to the groups, and then they were implemented. In addition, the weights of nude mice were regularly checked and recorded.

Control group: 100 μL of PBS was given three doses a week via the tail vein.

Compound **3(Y)** group: Compound **3**(**Y**) was prepared in DMSO at a concentration of 200 μM, and 20 μL was administered via the tail vein in three doses a week.

Compound **4(X)** group: Compound **4**(**X**) was prepared in DMSO at a concentration of 200 μM, and 20 μL was given three doses a week through the tail vein.

### 2.4 Toxicology Studies

Ethics committee approval for the study of toxicology studies was obtained from Ege University Animal Experiments Local Ethics Committee (Ethics Committee Number: 2020-029). The studies were carried out in Ege University ARGEFAR Pre-Phase Research Unit, where the care of experimental animals and controlled conditions were provided. To examine the toxicological efficacy of the two *N*-benzylisoindole-1,3-dione derivatives, a total of 88 CD1 mice, 44 male CD1 mice and 44 female CD1 mice, were used from 6–8 weeks old (15–25 g) albino mice. The mice were maintained under standard laboratory conditions (22 °C and 55% humidity), fed with water and standard pellet feed as forensic, and a 12 h night and 12 h day photoperiod was applied.

In the acute toxicity study, it was prepared from both *N*-benzylisoindole derivatives (**3** and **4**) as 200 μM. In total, 2 male and 2 female mice were administered intravenously once (100 μL) for items **3** and **4**, and the mice were observed for 14 days. For the subacute toxicity study, isoindole-1,3-dione derivatives were prepared as 200 μM (1st dose), 100 μM (2nd dose), and 50 μM (3rd dose). OECD Guideline 407 was used as the experimental design. Mice were grouped into three dose groups (10 mice in each group; 5 males and 5 females) and a control group. Subsequently, items **3** and **4** were administered intravenously. In the trial, intravenous administration was performed at a rate of 100 μL 3 times a week for a month. Control group mice were given physiological buffer (PBS) in the same conditions and volume of injection.

The mice were weighed every week during the application and their condition was monitored. When the study was completed (at the end of 4 weeks), the xylazine/ketamine solution prepared in a 2:1 ratio was administered intraperitoneally to the mice and sacrificed. Certain organs (heart, spleen, kidney, liver, and lungs) of mice were taken. In addition, the blood of the highest dose groups, 1st dose **3** and 1st dose **4**, and control group mice after sacrification were collected in lithium heparin tubes, and blood parameters; alkaline phosphatase (ALP), alanine transaminase (ALT), aspartate transaminase (AST), total bilirubin (TBIL) were examined.

### 2.5 Histopathological Analysis

At the end of the 28 day exposure period, organs (liver, kidney, heart, lung, and spleen) were removed under ketamine/xylazine anesthesia after intracardiac fixation with 4% paraformaldehyde, postfixed for 24 h and processed for paraffin embedding. Paraffin sections were cut into 5 μm thick slices in microtome (Leica RM 2145) and stained with routine hematoxylin and eosin (H&E).^13−15^ Histopathological evaluation was assessed by light microscopy (Olympus BX-51 light microscope, Olympus C-5050 digital camera) at a magnification of ×40.

## 3 Results

### 3.1 In Vitro Cytotoxicity

Our group recently determined that isoindole-1,3-dione derivatives are potential tyrosine kinase enzyme inhibitors and have antiproliferative effects against some cell lines according to MTT test results. It has been confirmed that the antiproliferative effects vary according to the nitrogen (N) atom and the groups attached to the ring. In addition, some compounds are known to exert cytotoxic effects against more than one cell line. In light of this information, we decided to examine the effects of cytotoxicities of *N*-benzylisoindole-1,3-dione derivatives against adenocarcinoma (A549-Luc) cells to expand these studies. Benzyl derivatives of isoindole-1,3-dione **3** and **4** were synthesized according to the method used in our previous work.^9^ First, the cytotoxic effects of both compounds against A549-Luc cells were examined. IC_50_ values of those drugs were calculated using the MTT activity assay. Cell viability of A549-Luc cells using two compounds was followed for 24, 48, and 72 h of incubation time. According to the results of these experiments, the optimum incubation period was determined as 48 h. The IC_50_ values of **3** and **4** were 114.25 and 116.26 μM, respectively (Table 2).

**Table 2:** IC_50_ Values of Compounds **3** and **4**

time (h)	compound **4** (**X**) (IC_50_ μM)	compound **3** (**Y**) (IC_50_ μM)
24	348.59	265.75
48	116.26	114.25
72	83.64	68.83

MTT assay results of *N*-benzyl derivatives compounds 3 and 4 showed that the degree of cytotoxicity in A549 cell lines is even greater than in HeLa cell lines. Based on the results obtained, we decided to study *N*-benzyl derivatives against adenocarcinoma (A549-Luc) cells in vivo and conducted in vivo animal experiments (preclinical study) with toxicological and histopathological studies to evaluate the potential of these compounds to be active ingredients.

### 3.2 In Vivo Therapy

To evaluate the in vivo cancer therapeutic potential of *N*-benzylisoindole-1,3-dione derivatives, three groups of five nude mice in each group were established: compound 3, compound 4, and the cancer control group. Male atypical nude mice aged 6–8 weeks are commonly used in in vivo xenograft tumor models. A549-luc lung cancer cells were injected into nude mice in all three groups, and tumor growth was induced with a xenograft model. For xenograft administration, 100 μL of 5 × 10^6^ A549-luc cell suspension was injected subcutaneously into nude mice. After cell application to nude mice, it was determined whether there was cancer or not by imaging with the IVIS device. After the cancer groups were formed, the treatment phase was started. Compound **4**(X) substance, compound **3**(Y), and cancer control groups were formed, five in each group. Each prepared sample was given to the animals by tail vein administration, three doses (as described in Section 2.3) per week, and the IVIS images taken at 5 min after luciferin (200 μL of 12 mg/mL stock administered subcutaneously) at certain time intervals were evaluated. Although no tumor was detected on the 15th day after cell implantation, the treatment was continued for two more weeks and whether the tumor recurred or not was determined by IVIS imaging. Survival nude mouse treatment was discontinued on day 30, nude mice were observed for 60 days, and survival and weight were determined. Weight changes of nude mice for 60 days are given in Table 3.

**Table 3:** Weight Changes of Nude Mice for 60 Days

	initially	3 days	7 days	10 days	15 days	20 days	30 days	40 days	50 days	60 days
**4** (1)	24.57	29.17	33.10	31.71	35.20	37.08	35.54	34.30	30.41	32.95
**4** (2)	22.94	26.62	30.76	30.85	32.46	Ex	Ex	Ex	Ex	Ex
**4** (3)	24.50	29.35	32.46	31.40	31.25	Ex	Ex	Ex	Ex	Ex
**4** (4)	23.01	24.80	25.32	27.13	28.34	Ex	Ex	Ex	Ex	Ex
**4** (5)	24.01	28.90	30.89	31.24	32.30	Ex	Ex	Ex	Ex	Ex
**3** (1)	22.18	26.25	29.26	30.26	32.30	33.78	32.96	32.30	33.12	33.37
**3** (2)	24.24	27.20	30.77	32.07	33.71	36.05	34.86	34.31	34.65	35.95
**3** (3)	23.58	26.25	28.50	28.52	31.02	32.60	30.90	30.81	30.91	32.39
**3** (4)	24.10	24.90	25.42	25.98	28.50	30.60	30.90	31.30	33.40	34.65
**3** (5)	22.14	23.54	24.87	25.89	26.43	28.70	29.40	30.31	31.25	32.60

As seen in Table 3, nude mice treated with substance **4** became Ex after 15 days. In subject number (1) of given compound **4**, there was an increase in weight up to the 20th day, although the weight decreased slightly later on. Cancer mice treated with substance **3** did not die, and survival was achieved for 60 days. After the 60th day, nude mice were sacrificed and the experiment was terminated.

As a control group, 100 μL of PBS was administered from the tail vein to five nude mice with cancer 3 times a week, and the tumor sizes detected after IVIS imaging are summarized in Table 4.

**Table 4:** Control Group Tumor Sizes

nude mice	tumor size initially	7 days	20–30 days
**1**	870.30	1100.20	sacrificed
**2**	424.83	900.30	sacrificed
**3**	721.13	1520.40	Ex
**4**	602.80	1810.50	Ex
**5**	590.60	1900.25	Ex

**Figure 3:**
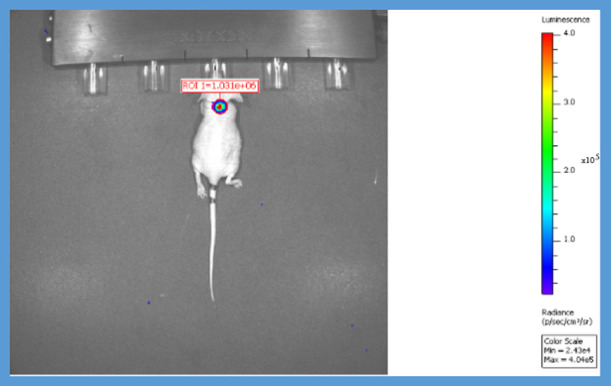
IVIS image of the control group.

When the tumor size of the control group was examined, the tumor size of the cancer animals, which were not treated with any treatment, increased in a short time and the whole control group was excluded from the experiment after the 20th–30th day (Figure 3).

**Figure 4:**
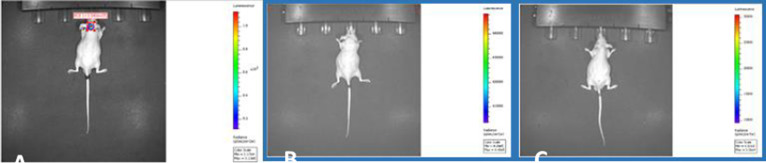
IVIS images of (A) control, (B) on the 7th day, and (C) on the 45th day after compound **4** (1) treatment.

The findings including tumor volumes during the initial and treatment period in nude mice treated with (1) of compound **4** are summarized in Table 5. IVIS images obtained by giving item **4** are given in Figure 4.

**Table 5:** Tumor Sizes for **4** (1) Compound

samples	tumor size initially (mm^3^)	7 days	15 days	45 days
**4** (1)	570.91	Tnd^a^	Tnd^a^	Tnd^a^
**4** (2)	403.71	Ex	Ex	Ex
**4** (3)	482.52	Ex	Ex	Ex
**4** (4)	1518.02	Ex	Ex	Ex
**4** (5)	1124.09	Ex	Ex	Ex

a Tnd (tumor not detected).

Nude mice coded **4** (2), **4** (3), **4** (4), and **4** (5) were found Ex in their cages after the 3rd dose by tail vein administration. The IVIS images taken by continuing the treatment of **4** (1) nude mice are given in Figure 4. As can be seen in Table 5 and Figure 4A–C, after treatment with substance **4**, the tumor was completely destroyed and tumor growth was not seen after the treatment was discontinued.

The findings including tumor volumes during the initial and treatment period in nude mice treated with IV (tail vein) with substance **3** are summarized in Table 6.

**Table 6:** Tumor Sizes for **3** (4) Compound^a^

sample	tumor size initially (mm^3^)	7 days	15 days	45 days
**3** (1)	404.93	Tnd	Tnd	Tnd
**3** (2)	301.26	Tnd	Tnd	Tnd
**3** (3)	424.93	Tnd	Tnd	Tnd
**3** (4)	731.33	Tnd	Tnd	Tnd
**3** (5)	600.97	Tnd	Tnd	Tnd

a Tnd (tumor not detected).

**Figure 5:**
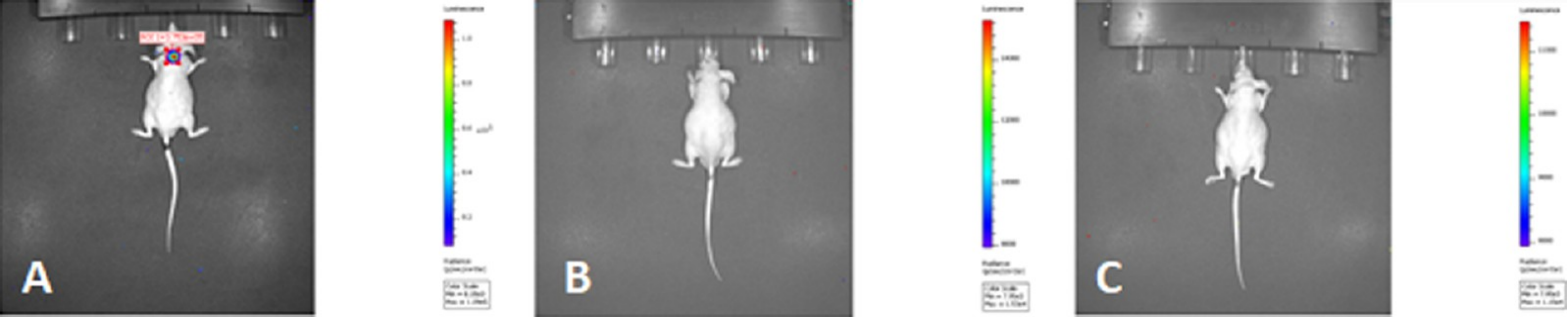
IVIS images of (A) control, (B) on the 7th day, and (C) on the 15th day after compound **3** (4) treatment.

As seen in Table 6, by giving item **3** (4), the tumor was completely destroyed after the treatment and survival was also provided. The IVIS images obtained for the **3** (4) coded nude mouse by giving compound **3** (4) are given in Figure 5.

As a result of the 3rd dose administration of compound **4** (1), it was decided to conduct 28 day repeated dose toxicology trials, since four nude mice were dead. For this purpose, (A) monitoring of survival and weight by giving three different doses of **4** to healthy CD1 mice (female and male) for 28 days, (B) performing biochemical analysis by taking blood from mice sacrificed after 28 days (for example, biochemical blood, hemogram, and determination of liver enzymes), and (c) histopathological evaluation for certain organs after sacrification was performed.

### 3.3 Toxicology Studies

**Figure 6:**
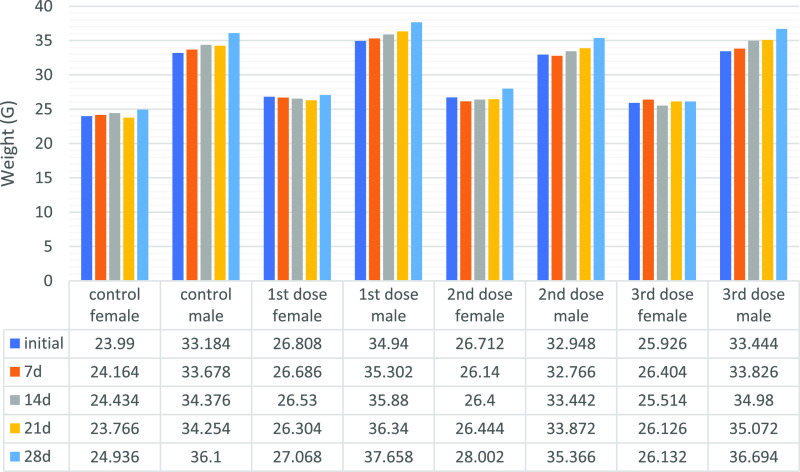
28 day subacute toxicity weight change graph of substance **4** groups: A graph was formed by taking the average weight of mice in the same group.

**Figure 7:**
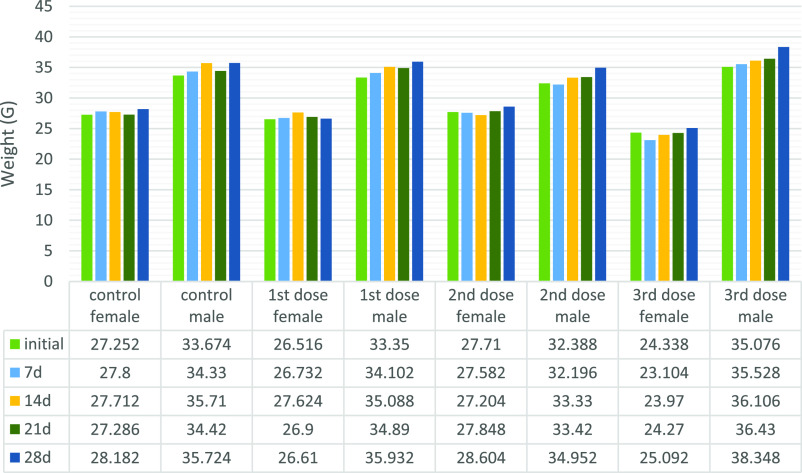
28 day subacute toxicity weight change graph of substance **3** groups: A graph was formed by taking the average weight of mice in the same group.

In the acute toxicity study, the effect of acute doses of substances **3** and **4** given to CD1 mice (2 females and 2 males) at a concentration of 200 μM over 14 days was examined. There were no abnormalities in the behavior of the mice for 14 days. In addition, there was no death in mice due to acute dosing of substances **3** and **4** after this trial, and repeated dose (subacute) toxicity studies were started. In the subacute toxicity study, mice were weighed before starting the experiment and grouped into three dose groups and a control group (5 females, 5 males, total 10 CD1 mice) for both *N*-benzylisoindole-1,3-dione derivatives 3 and 4. Then, an intravenous application of 100 μL was performed 3 times a week for 4 weeks. For 1 month, mice were controlled individually according to dose groups and sex. 28 day subacute toxicity weight change graphs are given in Figures 6 and 7.

Descriptions of the biochemical parameters performed in 1st dose **3** and 1st dose **4** group mice and control group mice are given in Table 7. According to the analysis of biochemical parameters, a slight increase was observed in ALT enzyme activity in the control group mice compared to dose groups **3** and **4**. It is known that while ALB level decreases in liver diseases, ALT and AST enzyme levels increase in liver diseases. When the AST levels of the control and dose groups were compared, an increase was found in both groups of mice, being more in the **3** group. When evaluated in terms of the amount of increase, the increase due to the **3** item is more than the increase related to **4**. In this case, histopathological evaluation was made to understand the full effect on the liver.

**Table 7:** Results of Biochemical Parameters

parameters	control group	200 μM 4a group	200 μM 3a group
ALT	44.5 ± 3.54	57.5 ± 0.71	60.5 ± 6.36
AST	76 ± 5.66	94.5 ± 3.54	114 ± 9.90
TBIL	0.08 ± 0.02	0.03 ± 0.03	0.02 ± 0.01
ALB	2.14 ± 0.20	2.63 ± 0.07	2.34 ± 0.05

### 3.4 Histopathological Studies

#### 3.4.1 Kidney Histopathological Results

Capsule structure and cortex–medulla discrimination were determined by examining the kidney structure of the mice belonging to the **3** (control) and **4** (control) groups. Glomeruli in the cortex were found in the normal histological structure.

**Figure 8:**
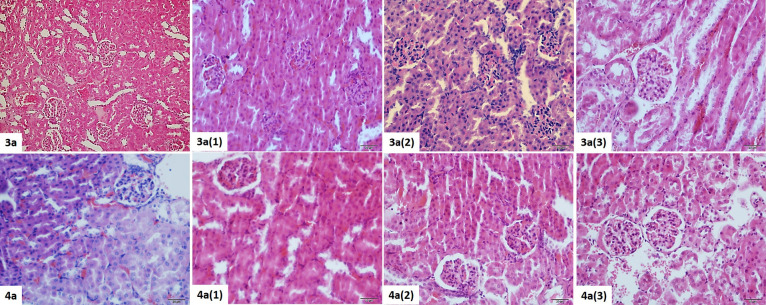
Histopathological view of the kidney tissue belonging to the **3** and **4** dose groups. H&E staining, ×40.

When groups **3** (1), **3** (2), and **3** (3) were examined, glomerular degeneration findings were observed mostly in 3a1 and less degeneration was detected at **3** (2) and **3** (3), consistent with the decrease in dose. In addition, degeneration and edema were observed in podocytes. Vacuolization and cystic dilatation in tubular structures were seen mostly at **3** (1). Peritubular vessel dilatation and congestion were observed most in 3a1 and moderately in **3** (2) and **3** (3). An increase in mesangial tissue and edema was observed in glomeruli in Group 3a1. In Group **3** (2), edema was found in mesangial tissue less than in Group **3** (3). Notably, Group **3** (1) had necrotic glomeruli in some areas. While the Bowman gap was normal in **3**, an increase in the gap was observed at **3** (3), **3** (2), and **3** (1) (maximum) consistent with a dose increase (Figure 8, Table 7).

In group **4** (control) normal liver structure was observed. Although histopathological changes were similar to the **4** group, findings were more severe in mice exposed to **3** agent (Figure 8, Table 8).

**Table 8:** Histopathological Changes in Kidney Tissues^a^

histopathological changes for the kidney	group **3–4**	group **3** (1)	group **3** (2)	group **3** (3)	group **4** (1)	group **4** (2)	group **4** (3)
degeneration in the renal corpuscles	N	+++	++	+	+++	++	+
degeneration and edema in podocytes	N	+++	++	+	+++	++	+
cystic degeneration of the renal corpuscles	N	+++	++	+	+++	++	+
dilatation and congestion in peritubular vessels	N	+++	++	+	+++	++	+
vacuolization in proximal tubules	N	+++	++	+	+++	++	+
epithelial changes in the distal tubules	N	+++	++	+	+++	++	+

a N, normal histology; +, low level of change; ++, moderate change; and +++, high level of change.

#### 3.4.2 Liver Histopathological Results

**Figure 9:**
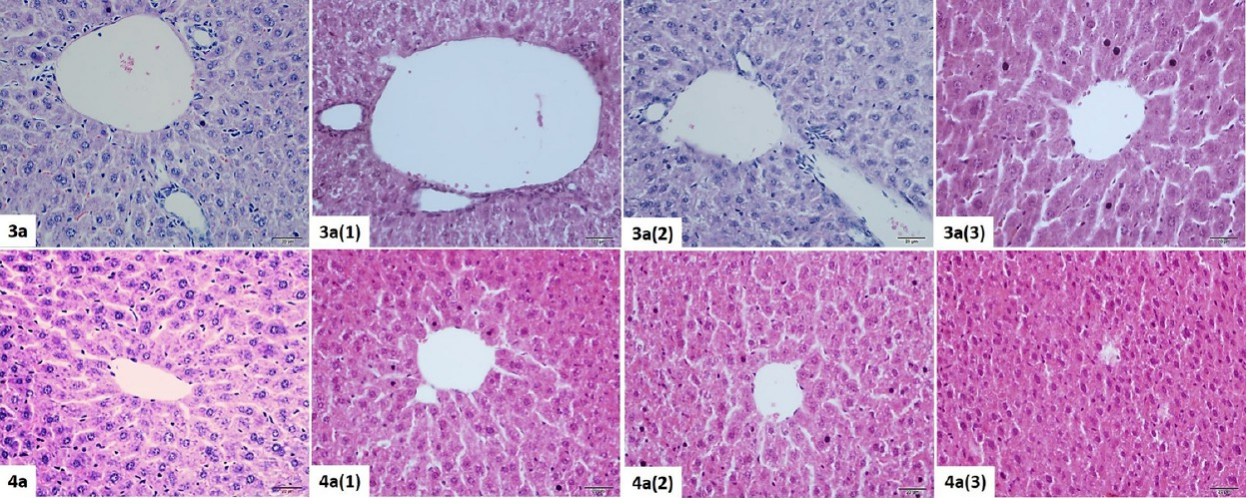
Histopathological view of the liver tissue belonging to the **3** and **4** dose groups. H&E staining, ×40.

In group **3**, the liver had a normal histomorphological structure. The organ was surrounded by the Glisson capsule, vena centralis located in the center of the liver lobule was observed in the normal histological structure. Remark cords radially emerging from the vena centralis were composed of hepatocyte cells located radially, and sinusoids were in their normal structures in lateral parts of hepatocytes. The portal vein, hepatic artery, and bile duct structures in the portal triad were observed naturally. Synozoidal structures in the basolateral parts of the hepatocytes were detected in normal width. It was determined that the sinusoidal structures and space of Disse in the **3** (1) group were wider than in the control group. Dilatation was seen in the portal triad, moderate dilatation was found in vena centralis in **3** (1) and minimal dilatation was found in **3** (2) and **3** (3). In addition, pycnotic hepatocytes were detected mostly in **3** (1). In the **3** (3) group, hepatocytes with pycnotic nuclei were found in only a few areas (Figure 9, Table 8). In group **4** (control), normal liver structure was observed. Although histopathological changes were similar to the **4** group, findings were more severe in mice exposed to **3** dose agents (Figure 9, Table 8).

It was found that the sinusoidal structures and space of Disse were larger than the control group, mostly in the **3** (1), then **3** (2) and **3** (3) groups. Dilatation was seen in the portal triad structure, while vena centralis had a high degree of dilatation in **3** (1) and moderate dilatation in **3** (2) and **3** (3) groups. Pycnotic hepatocytes were most frequently detected in the **3** (1) group, additionally even in the lowest dose, in the **3** (3) group, the number of pycnotic cells was found very high. In addition, intense intracytoplasmic edema and dispersion of glycogen granules in hepatocytes were found in the **3** (3) group. Although histopathological changes were similar to the **4** group, findings were more severe in mice exposed to **3** dose groups (Figure 9, Table 9).

**Table 9:** Histopathological Changes in Liver Tissues^a^

liver area	histopathological changes	group **3–4**	group **3** (1)	group **3** (2)	group **3** (3)	group **4** (1)	group **4** (2)	group **4** (3)
parenchyma	intracytoplasmic edema	none	+++	++	+	+++	++	+
	nuclear hypertrophy	N	+++	++	+	+++	++	+
	vena centralis dilatation	N	+++	++	+	+++	++	+
stroma	sinusoidal dilatation	N	+++	++	+	+++	++	+
	portal triad dilation	N	+++	++	+	+++	++	+

a N, normal histology; +, low level of change; ++, moderate change; and +++, high level of change.

#### 3.4.3 Lung Histopathological Results

When the lung parenchyma was examined, it was seen that the ductus alveolaris, terminal bronchiole, respiratory bronchiole, and alveolar structures were in normal histological appearance in group **4** (control). In experimental groups [**4** (1), **4** (2), and **4** (3)], there was widespread mononuclear infiltration in peribronchial and perialveolar areas where alveolar structures collapsed increasingly by the dose.

**Figure 10:**
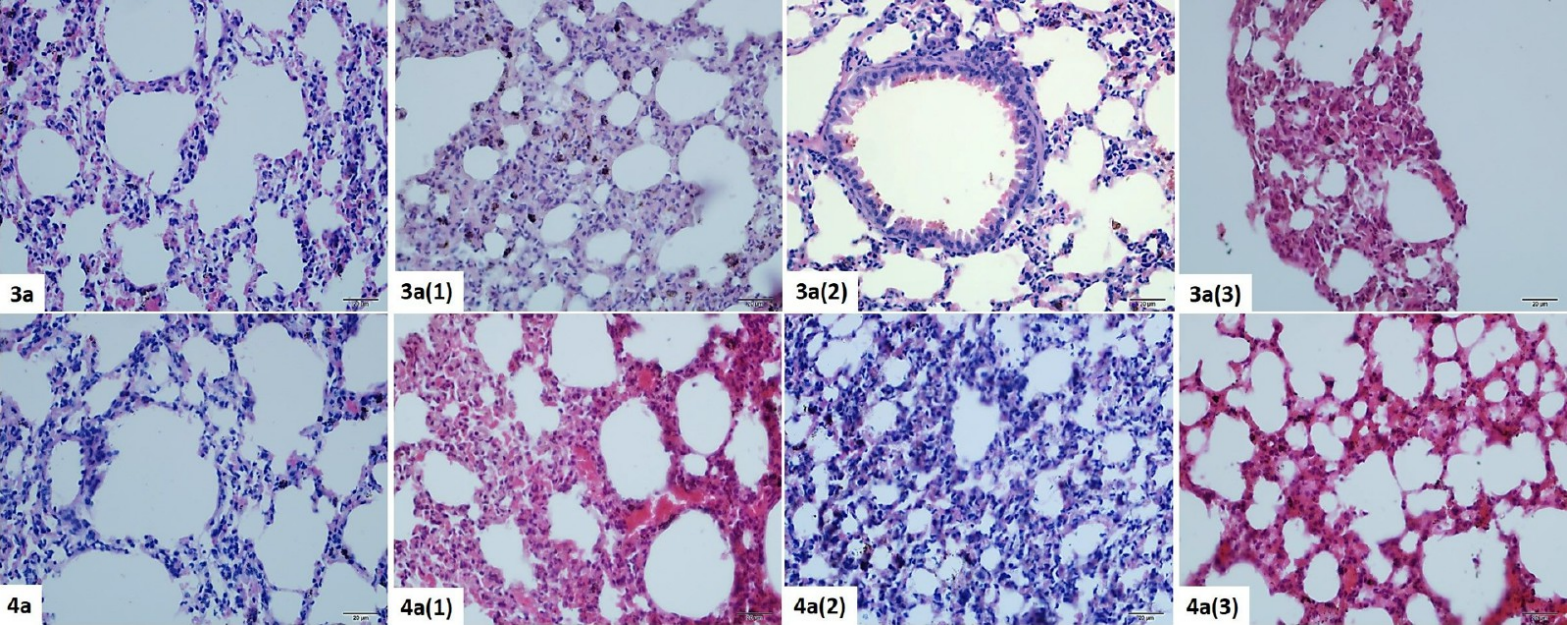
Histopathological view of the lung tissue belonging to the **3** and **4** dose groups. H&E staining, ×40.

Alveolar structures are commonly collapsed in **4** (1), in addition, there is intense mononuclear infiltration and edema in these areas. Notably, there was a high degree of vasodilation in vascular structures due to increase in the dose. Intense fibrosis was detected due to edema and inflammation in the lung tissue. Congestion was in very few areas in groups **4** (2) and **4** (3). In groups **4** (1), **4** (2), and **4** (3), epithelial length was higher than **4** (control), but the smooth muscle layer was thickened. Widespread mononuclear infiltration was detected in peribronchial areas. Alveolar thickening was observed to be the highest in Group 4a1 (Figure 10, Table 9).

Inflammation, edema, and fibrosis in **3** (1) were at a very high level compared to other groups. Unlike the **4a** agent, congestion was seen in mostly the **3** (1) group and in a very small area in **3** (2) and **3** (3) groups. Again, in the **3** (1) group, there was an obvious increase in alveolar dust cells (lung macrophages), which was not so obvious in **4** dose groups (Figure 10, Table 10).

**Table 10:** Histopathological Changes in Lung Tissues^a^

histopathological changes for the lung	group **3**	group **3** (1)	group **3** (2)	group **3** (3)	group **4**	group **4** (1)	group **4** (2)	group **4** (3)
subepithelial muscle thickening in terminal bronchioles	N	+++	++	+	N	+++	++	+
basement membrane thickening in the terminal bronchiole epithelium	N	+	++	+++	N	+++	++	+
mononuclear infiltration	N	+	++	++	N	++	++	minimal
dilatation and congestion in parenchymal vessels	none	minimal	+	+	none	+++	++	minimal
alveolar structures	none	+	++	+++	none	+++	+	+
alveolar wall thickness	N	++	++	+++	N	+++	++	+

a N, normal histology; +, low level of change; ++, moderate change; and +++, high level of change.

#### 3.4.4 Spleen Histopathological Results

**Figure 11:**
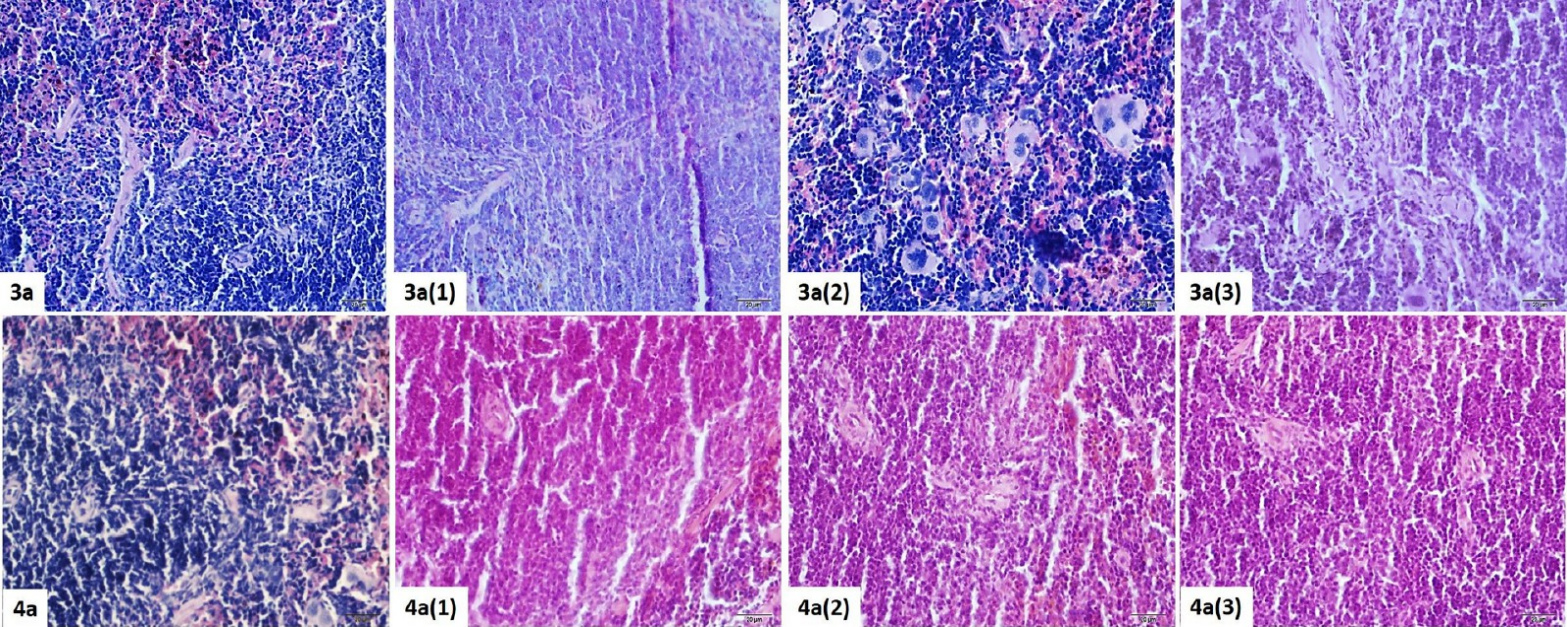
Histopathological view of the spleen tissue belonging to the **3** and **4** dose groups. H&E staining, ×40.

When the **4** (1), **4** (2), and **4** (3) experimental groups were examined, no histopathological degenerative finding was found at spleen tissue sections. However, a low degree increase in cell density compared to the **4** (control) group was detected. A moderate increase in cell density was detected in groups **3** (1), **3** (2), and **3** (3), similar to that in groups exposed to the **4** dose agents. However, there was a histopathological finding which was not observed in group **4**. Especially in the **3** (2) group, the presence of megakaryocyte-like cells in the peripheral blood, which were not seen under normal conditions, was quite remarkable. These cells were also present in the **3** (3) group less commonly. Interestingly, such cells were not found in sections of **3** (1), which was the highest dose administration group (Figure 11).

#### 3.4.5 Cardiac Muscle Histopathological Results

**Figure 12:**
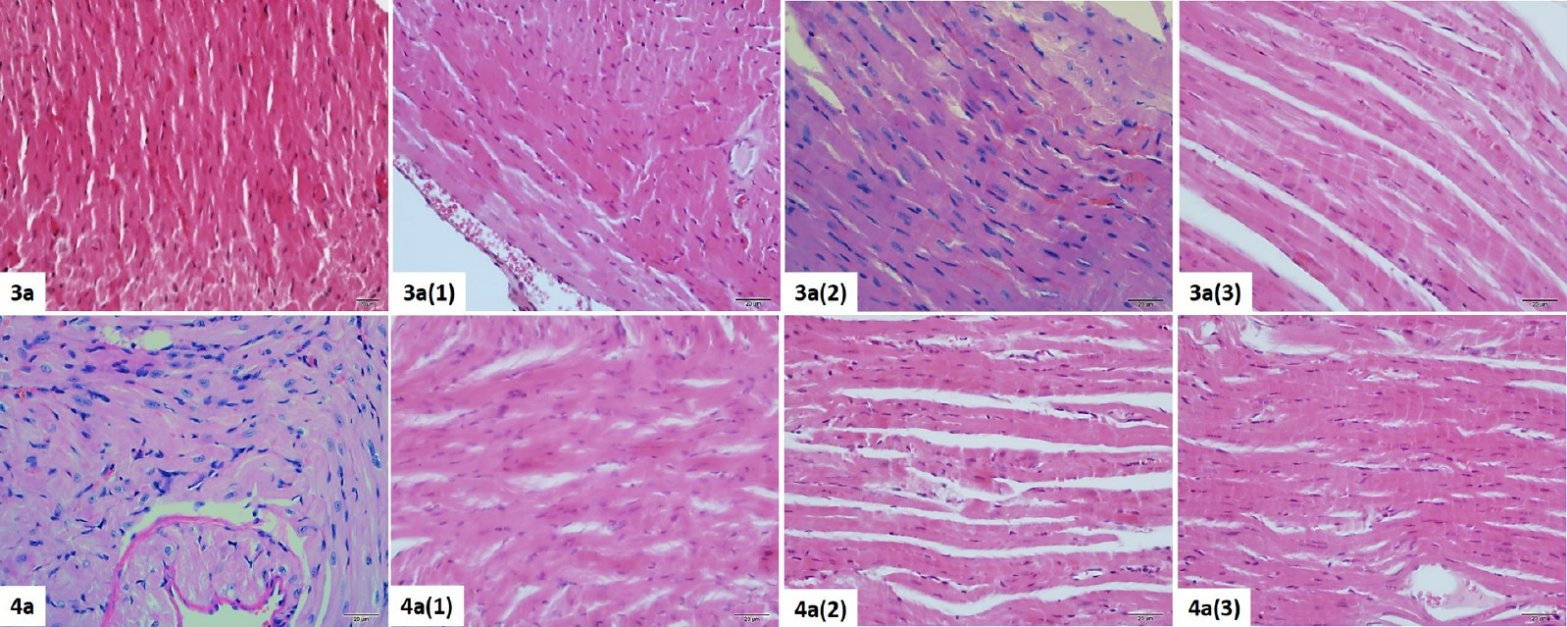
Histopathological view of the cardiac muscle tissue belonging to the **3** and **4** dose groups. H&E staining, ×40.

When cardiac muscle tissue sections are examined, a minimal increase was observed in the connective tissue between the muscle fibers, mostly in **4** (1), and there is a low/moderate level of deletion in the discus intercalaris. For **4** (2) and **4** (3), these findings decreased due to dose reduction and were close to normal histology (Figure 12). When the **3** groups were examined, it was seen that the increase in the connective tissue between the muscle fibers was at a higher level compared to the **4** groups. This increase was found mostly in the **3** (1) group and decreased in the other groups. Dilatation had been detected in the thin branches of the coronary. There was a moderate/high level of erasure in the discus intercalaris. Especially in the **3** (3) group, it was determined that the discus intercalaris structure started to be seen as normal again (Figure 12).

## 4 Discussion

The cytotoxic activities of two compounds **3** and **4** were evaluated in A549 cell lines. Based on the results of the study, we suggest that N-benzylisoindole derivatives might be good potential anticancer agents for the treatment of adenocarcinoma cancer due to their antiproliferative activities in cancer cells. In this context, preclinical studies were performed to reveal both compounds as potential anticancer agents. It was determined by animal experiments that both compounds cured tumors in mice with cancer. Examining the tumor size of the control group, it was found that the cancer animals which had not received any treatment had their tumor size increase rapidly within a few days, leading to the exclusion of the entire control group from the experiment after the 20th to 30th day. Four of the nude mice that were administered with Compound **4** (X) showed Ex after the third dose. After 15 days, the mice given Substance 4 had developed Ex. Subject 1 of Compound 4 had an increase in weight up to the 20th day, though it decreased slightly afterward. The cancer mice that received Substance 3 did not die and survived for 60 days. After the 60th day, the nude mice were sacrificed and the experiment was concluded. This result indicated that 28 day repeated-dose toxicology trials should also be conducted. Acute toxicity and subacute toxicity studies were performed. No anomalies and mouse deaths were observed in acute toxicity studies. In subacute toxicity studies, mice were weighed separately by compound type, dose groups, and sex. Biochemical analyses (ALT, AST, and ALB) were performed from the blood samples of the mice. Considering some changes in the values of the parameters, histopathological studies were carried out. Comparing the AST levels of the control and dose groups showed an increase in both groups of mice, with a higher increase in the **3** group. Evaluating the amount of increase, it was determined that the increase caused by **3** was greater than that of **4**. To gain a fuller understanding of the effect on the liver, a histopathological evaluation was then conducted. Histopathological evaluation was carried out by taking certain organs (such as the liver, kidney, heart, lung, and spleen) after sacrification. The observed histopathological changes are dose-dependent changes. For example, histopathological changes were greater in mice exposed to dose group **3**. The results obtained showed that these two compounds may be potential anticancer agents, especially for lung cancer. It is also predicted that these compounds have the potential to be used in phase-1 studies by dose adjustments.

## Notes

Approval for monitoring the cancer treatment potential of the synthesized structures in vivo conditions was taken by the Animal Ethics Committee of Ege University on 24.01.2018 with the number 2018-015. These experiments were conducted in accordance with all national or local guidelines and regulations.
